# Effects of Animal and Vegetable Proteins on Gut Microbiota in Subjects with Overweight or Obesity

**DOI:** 10.3390/nu15122675

**Published:** 2023-06-08

**Authors:** Claudia Di Rosa, Ludovica Di Francesco, Chiara Spiezia, Yeganeh Manon Khazrai

**Affiliations:** 1 Research Unit of Food Science and Human Nutrition, Department of Science and Technology for Sustainable Development and One Health, Università Campus Bio-Medico di Roma, Via Alvaro del Portillo 21, 00128 Roma, Italy; c.dirosa@unicampus.it (C.D.R.); ludovicadifra99@gmail.com (L.D.F.); chiara.spiezia@unicampus.it (C.S.); 2 Operative Research Unit of Nutrition and Prevention, Fondazione Policlinico Universitario Campus Bio-Medico, Via Alvaro del Portillo 200, 00128 Roma, Italy

**Keywords:** obesity and protein intake, proteins and gut microbiota, vegetable proteins and gut microbiota, animal proteins and gut microbiota

## Abstract

The gut microbiota plays a pivotal role in the balance between host health and obesity. The composition of the gut microbiota can be influenced by external factors, among which diet plays a key role. As the source of dietary protein is important to achieve weight loss and gut microbiota modulation, in the literature there is increasing evidence to suggest consuming more plant proteins than animal proteins. In this review, a literature search of clinical trials published until February 2023 was conducted to examine the effect of different macronutrients and dietary patterns on the gut microbiota in subjects with overweight and obesity. Several studies have shown that a higher intake of animal protein, as well as the Western diet, can lead to a decrease in beneficial gut bacteria and an increase in harmful ones typical of obesity. On the other hand, diets rich in plant proteins, such as the Mediterranean diet, lead to a significant increase in anti-inflammatory butyrate-producing bacteria, bacterial diversity and a reduction in pro-inflammatory bacteria. Therefore, since diets rich in fiber, plant protein, and an adequate amount of unsaturated fat may help to beneficially modulate the gut microbiota involved in weight loss, further studies are needed.

## 1. Introduction

Obesity is a rapidly growing multifactorial disease condition globally [1]. In fact, according to the World Health Organization (WHO), the global prevalence of this condition almost tripled between 1975 and 2016 and in that year, more than 1.9 billion adults were overweight including more than 650 million with obesity [2]. This condition is characterized by an excess of fat mass that negatively affects health. Obesity is classified according to Body Mass Index (BMI). When it is between 25.0 and 29.9 kg/m^2^, it is defined as overweight, while when it is >30.0 kg/m^2^, it is defined as obesity. A high BMI is a risk factor for non-communicable diseases such as type 2 diabetes, cardiovascular diseases, musculoskeletal and metabolic disorders, and certain types of cancer, resulting in a drastic decrease in quality of life and reduced life expectancy [3,4].

Gut microbiota consists of approximately 3.8 × 10^12^ microorganisms [4], mainly bacteria, archaea, and eukaryotes [5]. Normally, the gut microbiota plays several beneficial roles for the host, including involvement in carbohydrate and lipid metabolism, vitamin and amino acid synthesis, epithelial cell proliferation, protection from pathogens, and hormonal modulation [4]. An imbalance in microbial populations, called ‘dysbiosis’, is associated with a wide range of diseases including neurological disorders, inflammatory bowel disease (IBD), malnutrition, cancer, type 2 diabetes, and obesity [6]. Dysbiosis may also alter the functioning of the gut barrier and gut-associated lymphoid tissues (GALT) by allowing the passage of lipopolysaccharides (LPS), which activate inflammatory pathways that may contribute to the development of insulin resistance [7]. Furthermore, dysbiosis appears to be linked to alterations in intestinal motility, chronic low-grade inflammation, alterations in the enteric nervous system, and vagal afferent neurons, and typical symptoms of irritable bowel syndrome (IBS) such as abdominal pain, bloating, but also alterations in bowel movements [8]. The composition of the gut microbiota can be influenced by external factors such as diet, medication, the intestinal mucosa, the immune system, and the microbiota itself. Among these, diet plays a key role [9].

Many studies have been conducted to determine the best macronutrient composition for weight loss [10,11,12,13,14]. Various dietary patterns for weight loss have been studied, but among these, an important role is played by proteins. Proteins have been found to be more satiating than carbohydrates and fats due to the increased concentration of amino acids in the blood and hunger-inhibiting hormones: this can lead to weight loss through reduced calorie intake [11,12,13,14]. Furthermore, dietary protein increases total energy expenditure (TEE) because protein has a significantly higher diet-induced thermogenesis (TID) than carbohydrates and fats [11]. Furthermore, proteins are able to conserve fat mass loss during weight loss and a higher intake of proteins promotes weight loss maintenance [15,16]. Due to the preservation of fat mass, the decrease in the nocturnal metabolic rate (REE) is also preserved, contributing to weight loss [11]. Not only the amount of protein, but also the source, can be important for weight management. Proteins can be recognized from different sources: animal proteins from red and/or processed meat, poultry, fish, eggs, whey, and dairy products, and plant proteins from cereals, legumes, soy, nuts, pulses, fruits, and potatoes [17]. The present review aims to analyze the effects of animal and plant proteins on weight loss and the composition of the gut microbiota.

## 2. Search Strategy

A literature search was conducted to identify clinical studies that examined the effects of different macronutrients and dietary patterns on the gut microbiota in subjects overweight or affected by obesity. We searched for articles published until February 2023 to summarize the most recent findings in the PubMed database using the following keywords; ‘obesity and protein intake’; ‘protein and gut microbiota’; ‘plant protein and gut microbiota’; ‘animal protein and gut microbiota’. Original articles, reviews, and meta-analyses published in English on mice and humans were included. In our review, we considered studies with an amount of protein ≥ 20% of total daily energy or >1.3 g/kg body weight/day. Studies on normal-weight subjects, unpublished studies, articles not in English, or papers in which the amount of protein was not indicated were excluded. Possible eligible studies were assessed on the basis of the abstract and then included in the article according to the inclusion and exclusion criteria. Subsequently, a manual search through the citations of the included articles was conducted to identify further eligible work.

## 3. The Human Gut Microbiota: Focus on Subjects with Obesity

In recent years, the relationship between the human gut microbiota and obesity has been widely studied. Most of the bacteria inhabiting the gastrointestinal tract are classified into four main bacterial phyla: Firmicutes, Bacteroidetes, Proteobacteria, and Actinobacteria [8], while secondary phyla include Fusobacteria and Verrucomicrobia [18]. The highest bacterial population density resides in the colon and the number of microorganisms is approximately ten times the total number of cells in our bodies [8]. Of these, Bacteroidetes and Firmicutes constitute more than 90% of the total microbial population [19].

In the case of excess body weight, a reduced number and lower diversity of species in the gut microbiota have been found [20]. The reduction in microbial diversity, combined with an overgrowth of Proteobacteria (which are normally a potentially pathogenic phylum, present in low percentages), are key factors in dysbiosis [21]. In this condition, there is also a significant increase in Firmicutes and a reduction in Bacteroidetes [22]. Other scientific studies have shown an increase in *Lactobacilli* [23] and a decrease in *Bifidobacteria* and *Akkermansia muciniphila*. These species are generally associated with improved function of the intestinal mucosa through the production of anti-inflammatory metabolites such as short-chain fatty acids (SCFA) [24,25,26].

Consequently, if there is no adequate barrier integrity, components of Gram-negative bacteria such as lipopolysaccharide (LPS), an endotoxin, cross the intestinal barrier, and enter the blood circulation where they induce chronic, silent inflammation in various tissues, and organs (pancreas, liver, muscle, and adipose tissue) [1,27]. LPS leads to an inflammatory state due to the activation of the TLR4 receptor, which is present in most cells and macrophages, thus activating the cytokine expression cascade which induces the inflammatory response [28]. This LPS-induced endotoxemia state leads to increased fasting blood glucose and insulin levels, body weight (given by an increase in adipose tissue), inflammatory markers, and levels of triglycerides stored in the liver [29].

## 4. Dietary Patterns Suggested for Weight Loss

Diet modification plays a key role in the treatment of obesity. In the absence of other specific therapeutic indications, it should aim to reduce the initial body weight by approximately 10%, especially in the case of overweight or stage I/II obesity (BMI between 30.0 and 39.9 kg/m^2^), within a reasonable time frame of four to six months [30]. Only in the case of stage III obesity (BMI > 40.0 kg/m^2^) is it necessary to lose more than 10% of the initial body weight, although this is sometimes difficult to maintain in the long term [30].

Available weight loss diets include differences in energy restriction, macronutrient composition, foods, and dietary patterns [31]. In recent years, many dietary patterns have been tested for their effectiveness in preventing and treating obesity: the low-calorie diet (LCD) with 800–1800 kcal/day and the very low-calorie diet (VLCD) with <800 kcal/day, the low-fat diet (<30% of total kcal/day), the low-carbohydrate diet (<20–45% of total kcal/day, with 60–130 g of carbohydrate/day), the high-protein diet (>20 [30]–25% of total kcal/day or >1.3 [32,33]–1.6 g protein/kg ideal body weight [33,34]), the low-calorie low-glycemic diet, the low-calorie DASH diet, the low-calorie Mediterranean diet [10,33,35,36], and the ketogenic very low-calorie diet (VLCKD) (<800 kcal with <50 g/day of carbohydrates, <30–40 g/day of lipids and between 0.8–1.5 g/kg ideal body weight of protein/day) [35,37,38]. In summary, there are several dietary approaches that can lead to the recommended weight loss. In fact, although all of these diets help to reduce energy intake, none has been shown to be more effective than another in producing clinically significant weight loss [10].

Whatever the dietary pattern, the importance of the protein source must also be discussed with regard to weight loss. Although some authors have found that there is no difference between animal and vegetable protein consumption, other authors have stated that animal protein consumption leads to long-term weight gain compared to vegetable protein consumption [17,39]. Among animal proteins, red meat, processed meat and poultry were found to be associated with an increase in body weight, whereas fish, and dairy products showed no effect on body weight changes [40]. On the other hand, plant proteins show a greater protective effect against obesity than animal proteins [41]. Currently, there are no indications that higher intakes of plant proteins and lower intakes of animal proteins influence body weight maintenance after weight loss, but higher intakes of plant proteins from non-grain products (legumes, soy, nuts, vegetables, potatoes, and fruit) have been associated with body weight maintenance [17]. In fact, 25–43 g/day of plant proteins or more than 50% plant proteins/day of total protein intake was associated with a reduction in body weight, BMI and waist circumference [42].

## 5. Effect of Macronutrients on Gut Microbiota

Dietary factors play a pivotal role in gut microbiota composition changes [43,44]. The effect of different macronutrients, such as digestible and non-digestible carbohydrates, fats and proteins, is discussed in the literature. The most recent challenge is to identify the specific nutrient or dietary pattern that can promote the growth of beneficial gut microbiota populations leading to the production of bioactive metabolites [44].

### 5.1. Carbohydrates

Carbohydrates are the most studied components of the diet for their ability to modify the gut microbiota [44]. They can be classified into digestible and non-digestible. Digestible carbohydrates, which can be degraded in the small intestine, include both sugars (glucose, fructose, sucrose, and lactose) and starches, whereas non-digestible carbohydrates, which cannot be degraded enzymatically in the small intestine include dietary fibers and resistant starch. The former, after their degradation, release glucose into the blood stream. In two studies, ingestion of fruit sugars (glucose, fructose, and sucrose) led to an increase in *Bifidobacteria* and a reduction in the relative abundance of Bacteroides [45,46]. Another study showed that the addition of lactose to the diet led to the same bacterial changes and a decrease in the abundance of *Clostridia* [47]. The latter, on the other hand, arrive intact in the colon where they are fermented by the gut microbiota [48] producing compounds known as short-chain fatty acids (SCFA) that can benefit the health of the host by stimulating the growth of certain microorganisms [49]. This occurs mainly with the ingestion of soluble fibers. The bacterial phyla most affected by dietary fibers are Firmicutes and Actinobacteria [50]. Soluble fibers include inulin, hemicellulose, β-glucan, gums and mucilages, fructans (inulin and fructo-oligosaccharides (FOS)), galacto-oligosaccharides (GOS), dextrins, polydextrins, and resistant starch [51]. In a study by Cotillard et al., a diet with a high intake of these fibers in 49 subjects with obesity resulted in an increase in the gene richness of the microbiota [52]. Considering that the daily fiber intake recommended by the 2015–2020 American Dietary Guidelines Recommendations is an amount of 14 g of fiber per 1000 kcal consumed [53]. Therefore, in a 2000 kcal diet, the recommended fiber amount is 28 g/day. A low fiber intake is considered when it is under 10 g/day and it is considered to be a dangerous risk factor for the potential pathogenesis of bowel diseases [54].

Several studies have shown how dietary fibers can influence the composition of intestinal *Bifidobacteria*, resulting in a bifidogenic effect. A diet rich in whole grains and wheat bran resulted in an increase in intestinal *Bifidobacteria* and *Lactobacilli* [55,56], with a peculiar effect of fructans and GOS [57]. Costabile et al. conducted a study on 32 healthy volunteers with a BMI between 20 and 30 kg/m^2^ in which subjects were required to consume 48 g of whole grain cereals (WG) or wheat bran (WB) for two three-week periods, separated by a two-week washout period. The result was that the WG group showed a higher amount of *Bifidobacteria* and *Lactobacilli* and, in contrast, no differences in the amount of SCFA between the two groups [55].

A study investigated the bifidogenic effect of inulin (a fructan) on the gut microbiota. It was conducted by Birkeland et al. on 25 subjects with BMI < 40 kg/m^2^ suffering from type II diabetes and analyzed the effect of 16 g of inulin-type fructans (a mixture of oligofructose and inulin) or 16 g of placebo (maltodextrin) for six weeks, in randomized order, on the gut microbiota. The results highlighted the bifidogenic effect of inulin, especially the increase in *Bifidobacterium adolescentis* and SCFAs [58].

A randomized controlled pilot study conducted by Sheflin et al. on 29 overweight and with obesity volunteers examined the effects of a 28-day supplementation of fiber-rich stabilized rice bran (30 g/day) or cooked sea bean powder (35 g/day) on the gut microbiota. Subjects receiving rice bran experienced a significant decrease in the Firmicutes/bacteroidetes ratio and an increase in SCFAs [59]. Several studies investigated the effect of resistant starch (RS) on the composition of the gut microbiota. RS and whole grain barley led to an increase in the abundance of *Ruminococcus*, *Eubacterium rectale*, and *Roseburia* in three different studies [60,61,62]. In the study by Walker et al. [60], 14 overweight men were successively given a control diet, a high resistant starch (RS) (26 g/day of resistant starch), or non-starch polysaccharide (NSP) diet (42 g/day of total non-starch polysaccharides) and a reduced-carbohydrate weight loss (WL) diet for 10 weeks. The RS diet resulted in an increase in *Ruminococcus bromii*, representing 17% of total gut bacteria compared to 3.8% on the NSP diet. The RS diet also resulted in an increase in *Eubacterium rectale* (10.1% of total gut bacteria). The RS and WL diets showed an increase in *Oscillibacter* and the WL a decrease in *Eubacterium rectale* and *Collinsella aerofaciens* [60]. A randomized crossover study by Abell et al. examined a dietary intervention on 46 overweight volunteers assigned to two groups: one received an RS2-rich diet (22 g/day of RS) and the other a low-RS2 diet (1 g/day of RS). The researchers found that the gut microbiota of the group that followed an RS2-rich diet showed an increase in *Ruminococcus bromii*, *Faecalibacterium prausnitzii*, and *Eubacterium rectale*. They also observed that *Eubacterium rectale* was positively correlated with SCFAs production, especially butyrate [63] (Table 1).

### 5.2. Fats

The type of fat is also a strong determinant of gut microbiota composition and inflammation. There are studies in the literature concerning the effects of fat on the gut microbiota. Diets rich in saturated fats (lard) are associated with increased white adipose tissue (WAT) inflammation and metabolic disease, whereas diets rich in polyunsaturated fatty acids (fish oil) may counteract inflammation by promoting a lean and metabolically healthy phenotype [64]. Several studies have shown that not only dietary factors but also microbial factors can directly contribute to WAT inflammation, as gut microbial factors can be ligands of Toll-like receptors (TLRs) and trigger inflammatory signaling [29]. Several human studies have found that a high-fat diet increases total anaerobic bacteria and Bacteroides [65,66,67]. High-fat diets (HFD) typically contain about 32–60% of calories from fat [68].

Wan et al. observed in a six-month randomized controlled trial on 217 overweight subjects that a high-fat diet (40%) was correlated with an increase in *Alistipes*, Bacteroides, and LPS and a reduction in *Faecalibacterium* and total SCFAs concentration, compared to a low-fat diet (20%) [69].

Furthermore, Fava et al. found that consumption of a low-fat diet (28% of total daily energy from fat) increased the fecal abundance of *Bifidobacterium* in 88 overweight subjects [66] and they showed that four-week consumption of a high-saturated-fat diet (38% of total daily energy from fats, of which 18% were saturated fats) increased the relative abundance of *Faecalibacterium prausnitzii*, whereas a high-monounsaturated-fat (38% of total daily energy from fats, of which 20% of which were monounsaturated fats) diet did not shift the relative abundance of any bacterial genera but had an overall reduction in total bacterial load [66]. A study conducted by Rajkumar et al. on 60 overweight adults showed that supplementation with one capsule per day of omega-3 polyunsaturated fats (PUFA) (containing 180 mg of EPA and 120 mg of DHA per capsule) for six weeks did not lead to a significant change in the composition of the gut microbiota [70]. In another study by Balfegò et al., 35 patients overweight and with obesity were randomized into two groups: one followed a standard diet and the other a diet enriched with 100 g of sardines for five days per week for six months. The group that followed the enriched diet showed a reduction in the Firmicutes/Bacteroidetes ratio and an increase in the Bacteroides/Prevotella ratio, compared to baseline [71]. Jian et al. conducted a study on 38 overweight and with obesity subjects, analyzing the effect of an excess of 1000 kcal/day in a diet rich in saturated (86% of total daily energy from fat, of which 76% were saturated) or unsaturated fats (91% of total daily energy from fat, of which 79% were unsaturated) on the gut microbiota. The excess of saturated fat led to an increase in Proteobacteria, while the unsaturated diet increased butyrate-producing bacteria [72]. A randomized controlled trial (RCT) conducted by Vijay et al. compared the effects on gut microbiota composition of a six-week dietary intervention of 500 mg omega-3 supplementation with 20 g inulin supplementation in a population of 69 subjects with a BMI between 20 and 39.9 kg/m^2^. The omega-3 supplementation resulted in a significant increase in *Coprococcus* spp. and Bacteroides spp. and a significant decrease in fatty liver associated with *Collinsella* spp. [73]. Another RCT on 76 adults with overweight/obesity, conducted by Bratlie et al., examined the effects of eating salmon, cod (both in the quantity of 750 g/week, accordingly five dinners per week consisting in 150 g of salmon or cod fillet), or total fish exclusion (control group) for eight weeks on the composition of the gut microbiota. The results showed that the salmon group had a lower abundance of Bacteroidetes, Clostridiales, and a higher abundance of Selenomonadales, compared to the control group. The cod group showed similar results to the salmon group [74]. A study by Telle-Hansen et al. on 17 subjects with a BMI comprised between 18.5 and 27 kg/m^2^ showed differences in the composition of the gut microbiota after consumption of saturated fatty acid (SFA) (29.9 g of SFAs/day) or polyunsaturated fatty acid (PUFA) (26.4 g of PUFAs/day) products for three days. Lachnospiraceae and *Bifidobacterium* spp. increased significantly after the intervention with PUFA compared to that with SFA [75] (Table 2).

### 5.3. Proteins

The effects of dietary proteins on the gut microbiota are little studied but were first described in 1977 [44]. In studies investigating the impact of dietary proteins on the composition of the gut microbiota, the consumption of different protein sources is taken into account. Most studies have shown that protein consumption is positively correlated with total microbial diversity [52,67,76,77]. Only a few studies have been conducted specifically on the effect of plant proteins on modulating the gut microbiota. Consumption of whey and pea protein extracts has been reported to increase commensal *Bifidobacterium* and *Lactobacillus*. Pea protein has also been observed to increase intestinal SCFA levels, which are considered to be primary anti-inflammatory compounds for intestinal barrier integrity [78,79]. In a double-blind study on overweight subjects, Beaumont et al. investigated the influence of the amount and source of dietary protein on the production of metabolites in the gut microbiota. Participants received a 15% of their total energy daily intake from dietary supplements of plant protein (soy protein isolate) for three weeks for the soy protein group—SOY group), animal protein (milk protein isolate enriched in micellar casein for the casein group—CAS group), or digestible carbohydrates as an isocaloric control (maltodextrin) for the maltodextrin group (MD group). Analysis of the fecal and mucosa-associated microbiota revealed no significant differences in bacterial composition and diversity between the dietary intervention groups. On the contrary, they induced a shift in bacterial metabolism towards amino acid degradation with different metabolite profiles depending on the protein source: the CAS group resulted in an increased concentration of the bacterial metabolite derived from isoleucine, 2-methylbutyrate, while the SOY group had a higher relative concentration of several bacterial metabolites derived from AA such as valerate, phenylacetate, and tyramine. Acetoin production was only detected in the SOY group. These data, therefore, suggest that a high-protein diet (with soy or casein supplementation) may increase luminal concentrations of beneficial and harmful compounds [80].

On the other hand, several clinical studies have been conducted on the effect of animal protein in modulating the gut microbiota. Hentges et al. demonstrated how a diet high in beef, with an intake of 176 g/day of protein, led to a decrease in the abundance of *Bifidobacterium adolescentis* and an increase in *Bacteroides fragilis* and *vulgatus*, compared to a meat-free diet with an intake of 90 g/day of protein [81].

In a study by Romond et al., 15 mL supplementation of whey protein concentrate and fermented with *Bifidobacterium breve* led to a decrease in the pathogens *Bacteroides fragilis* and *Clostridium perfringens* [82]. A study by Sun et al. in overweight or with obesity subjects showed that a supplementation with 15.2 g/day whey protein or 16.8 g/day whey protein hydrolysate did not lead to significant changes in the composition of the gut microbiota, compared to a control group with a protein intake of 50 g/day [83]. A 12-week double-blind study conducted by Reimer et al. on 125 adults with overweight or obesity showed that the daily intake of two isocaloric whey protein bar (with 5 g of whey protein each) did not lead to a change in the gut microbiota compared to the control group [84]. Another study, conducted by Mitchell et al. on 31 elderly subjects with a BMI comprised between 20 and 35 kg/m^2^, looked at daily protein intake and showed that consuming a diet with twice the recommended dietary allowance (2RDA) of protein (from food sources) for 10 weeks did not result in significant differences in proteolytic microbiota populations or metabolites derived from protein fermentation, compared to the same diet containing the recommended dietary allowance (RDA) of proteins [85]. In general, focusing on animal protein consumption has been shown to increase the abundance of *Bacteroides*, *Alistipes*, and *Bilophila* [52,67,76]. Russell et al. found that 17 subjects with obesity on a high-protein/low-carbohydrate diet (137 g/day protein, 143 g/day fat, and 22 g/day carbohydrates) showed a reduced abundance of *Roseburia* and *Eubacterium rectale*, resulting in a decreased percentage of butyrate in feces [86]. This low abundance of *Roseburia* and other butyrate-producing bacteria was also found in a study of overweight IBD patients [87].

Data from Clarke et al., who conducted a study on 40 overweight male elite rugby players, demonstrated the importance of a varied diet, especially protein consumption (22% of total daily energy intake) associated with exercise, in changing the composition of the gut microbiota. In this study, protein consumption was positively correlated with high microbial diversity (22 distinct phyla) and athletes with lower BMI had significantly higher levels of *Akkermansia* [77], which had previously been shown to be inversely correlated with obesity in overweight and with obesity subjects [24,88] (Table 3).

These results underline the importance of dietary protein and exercise in positively modulating the gut microbiota. Further studies are needed to investigate the impact of protein on modifying the gut microbiota.

## 6. Effect of Different Nutritional Protocols According to the Amount and the Kind of Proteins on Gut Microbiota

Several diets, including Western, Mediterranean, plant-based (vegetarian/vegan), and the very low-calorie ketogenic diet (VLCKD), have been studied for their ability to modulate the composition of the gut microbiota. In general, a diet that includes the consumption of protein and animal fats correlates with the Bacteroides-dominated enterotype. Conversely, a diet rich in carbohydrates, especially fermentable fiber, is associated with the Prevotella-dominated enterotype [89].

### 6.1. The Western Diet

Considering the effects of dietary patterns, the Western-style diet or various combinations of high-fat (HF) diets, such as HF-high in sucrose and HF-low in plant polysaccharides, have shown negative effects on the composition of the gut microbiota in both animals and humans, often with conflicting results [43]. In several studies, the Western diet, characterized by a high intake of protein from animal sources, saturated fat, simple sugars, and a low dietary fiber content (from vegetables and whole grains), led to a significant decrease in the number of total bacteria and beneficial species such as *Bifidobacterium* and *Eubacterium* [63,66]. The Western diet has also been associated with the production of cancer-promoting nitrosamines [90]. In another study conducted by O’Keefe on 20 African-Americans and 20 rural Africans with a BMI between 18–35 kg/m^2^, after a two-week high-fat (52% energy) and low-fiber (12 g/day) Western-style dietary intervention, the abundance of *Fusobacterium nucleatum*, which is prevalent in human colorectal cancer, increased [91]. David et al. conducted a dietary intervention study on 10 subjects with a BMI between 19 and 32 kg/m^2^. The intervention consisted of eating an animal-based diet consisting of meat, eggs, and cheese (69.5% fat, 30.1% protein and almost no fiber) for five days versus a plant-based diet consisting of grains, legumes, fruit, and vegetables (22% fat and 10% protein and 25.6 g fiber/1000 kcal). The animal-based diet resulted in an increase in the abundance of bile-tolerant microorganisms (*Alistipes*, *Bilophila* and *Bacteroides*) and a decrease in the levels of Firmicutes metabolizing plant polysaccharides in the diet (*Roseburia*, *Eubacterium rectale*, and *Ruminococcus bromii*) [76]. Reddy et al. studied the effects of consuming a Western mixed high-meat diet consisting in a consumption of 454 g/day of beef, pork, lamb, and/or chicken (23% protein, 45% fat, and 32% carbohydrates) for four weeks and then the effect of switching to a meat-free diet (20% protein, 30% fat and 50% carbohydrates) on the gut microbiota. During the period of consumption of a mixed Western diet with a high meat content, an increase in anaerobic Bacteroides, Bifidobacterium, Peptococcus, and Lactobacillus was found compared to the meat-free diet [67]. Cotillard et al. conducted a study on 38 with obesity and 11 overweight people who were invited to consume a high-protein diet for 12 weeks with 35% of daily energy coming from proteins, 25% from lipids, and 44% from carbohydrates. The dietary intervention increased the gene richness of the gut microbiota, but reduced Bifidobacterium and *Eubacterium rectale* [52] (Table 4).

### 6.2. The Mediterranean Diet

The Mediterranean diet has been considered a balanced and healthy diet to reduce the risk of noncommunicable diseases: in fact, it has been recognized by UNESCO as a World Heritage Site [92]. It is characterized by a content of beneficial fatty acids, rich in monounsaturated (from extra virgin olive oil), and polyunsaturated (from fish, nuts, and seeds) fatty acids, polyphenols, and other antioxidant compounds, rich in fiber and characterized by a low glycemic index of carbohydrates, and a relatively higher intake of vegetable protein than animal protein. It is characterized by moderate consumption of fish, poultry, and red wine and lower consumption of red and processed meats, dairy products, and sweets [93].

In the clinical trial by Rinott et al., authors found that a six-month intervention of a Green Mediterranean diet of 1500–1800 kcal/day for men and 1200–1400 kcal/day for women (rich in vegetables, with poultry and fish replacing beef and lamb intake, with a restriction of processed and red meats, with a daily intake of 28 g of walnuts, and with 3–4 cups per day of green tea and 100 g per day of frozen cubes of *Wolffia globosa* (Mankai strain), as a green smoothie for dinner) was associated with Prevotella enrichment, increased degradation of branched-chain amino acids (BCAAs), reduced *Bifidobacterium* genus and BCAA biosynthesis. The reduced abundance of *Bifidobacterium* and increased abundance of Ruminococcaceae in this study were also significantly associated with weight loss [94].

In another clinical study, conducted by Pagliai et al., 23 overweight/with obesity omnivores were randomly assigned to either a Mediterranean diet or a plant-based diet for three months. The two dietary interventions consist of 50–55% of total daily energy from carbohydrates, 25–30% from fat, and 15–20% from proteins. The Mediterranean diet was characterized by the consumption of all food groups including meat and meat products, poultry, and fish. The Vegetarian diet was characterized by abstinence to consume meat and meat products, poultry, fish, seafood, and flesh from any other animal, but included eggs and dairy products. Both dietary interventions did not produce significant changes in gut microbiota composition at the phyla or family level, but at the genus level, a change was observed. In fact, the Mediterranean diet significantly increased the abundance of *Enterorhabdus* and *Lachnoclostridium* and decreased *Parabacteroides*. On the other hand, the vegetarian diet significantly increased the abundance of *Anaerostipes* and *Streptococcus* and decreased *Clostridium* and *Odoribacter*. The Mediterranean diet resulted in a 10% increased trend in propionic acid compared with the lower trend caused by the vegetarian diet (−28%). The change in SCFAs was correlated with a decrease in the abundance of some inflammatory cytokines, such as VEGF, MCP-1, IL-17 and IL-12, only after the Mediterranean diet: this appears to mediate the beneficial anti-inflammatory and protective roles of the Mediterranean diet [95]. An increase in butyrate-producing bacteria, which are one of the actors of anti-inflammatory effect, could provide a possible antitumor action. Indeed, butyrate acts as a histone deacetylase (HDAC) inhibitor, blocking the growth of colorectal cancer cells [96]. Gutierrez-Diaz et al. identified higher levels of *Bacteroidetes*, Prevotellaceae, and *Prevotella* and lower presence of Firmicutes and Lachnospiraceae in 31 overweight subjects with a high Mediterranean Diet adherence score (MDS score > 4) calculated by the Mediterranean Diet Score. The median daily consumption of eight MDS components contributed one point to the total score: high consumption of cereals (including potatoes and bread), legumes, vegetables, and fruits, moderate consumption of ethanol, high ratio of mono-unsaturated/saturated lipids, low consumption of meat and meat products, and of milk and dairy products. Therefore, the total MDS ranged from 0 (lowest adherence to traditional MD) to 8 (highest adherence), and the cut-off for stating high adherence is an MDS ≥ 4. The group with high adherence to the Mediterranean diet (≥4) was characterized by 48 g/day of proteins (of which 32 g/day are from animal sources and 15 g/day are from vegetable one), 103 g/day of carbohydrates and 40 g/day of fats, compared to a 51 g/day of proteins (of which 37 g/day are from animal source and 13 g/day from vegetable ones), 96 g/day of carbohydrates, and 43 g/day of fats of the lower adherence group [97]. In an eight-week RCT conducted by Meslier et al., 82 healthy overweight and with obesity subjects with habitually low fruit and vegetable intake and sedentary lifestyle were enrolled to assess the relative changes in the gut microbiota. Forty-three subjects followed an isocaloric Mediterranean diet (MedD) and 39 maintained their usual diet (ConD). The MedD group after the eight-week intervention resulted in a total amount of about 63 g/day of proteins (of which 37 g/day are from vegetable sources and 26 g/day from animal sources) with a fiber intake of about 31 g/day, whereas the ConD group followed for eight weeks their habitual diet with low intake of vegetable and fruits with a total amount of about 68 g/day of proteins (of which 20 g/day are from vegetable sources and 48 g/day are from animal one), and a fiber intake of about 13 g/day. The Mediterranean diet intervention increased levels of the liver-degrading *Faecalibacterium prausnitzii* and genes for microbial carbohydrate degradation related to butyrate metabolism [98] (Table 5).

### 6.3. The Plant-Based Diets

Vegetarian and vegan diets are rich in plant-derived foods, fermentable fiber, and plant protein. The study conducted by Pagliai et al., reviewed above, found that following a vegetarian diet (VD) or a Mediterranean diet (MD) for three months significantly increased the abundance of *Anaerostipes* and *Streptococcus* and decreased *Clostridium* and *Odoribacter* [95]. Kahleova et al. conducted a study on 168 overweight and obesity patients randomly assigned to a low-fat vegan diet (*n* = 84) or a control group (*n* = 84) for 16 weeks. The vegan group was asked to follow a low-fat vegan diet consisting of vegetables, grains, legumes, and fruits. They were asked to avoid animal products and added oils. Daily fat intake was limited to 20–30 g, and vitamin B12 was supplemented (500 µg/day). Subjects in the control group were asked to maintain their usual diet, which included animal products, for the duration of the study. The control group resulted in consuming 1700 kcal/day, 72 g/day of fats, 196 g/day of carbohydrates, 69 g/day of proteins (of which 39 g/day come from animal sources and 29 g/day from vegetable ones), and 23 g/day of fibers compared to 1300 kcal/day, 24 g/day of fats, 236 g/day of carbohydrates, 43 g/day of proteins (of which 1 g/day come from animal source and 42 g/day from vegetable one), and 33 g/day of fibers consumed by the vegan group. The relative abundance of *Faecalibacterium prausnitzii* increased in the vegan group and was negatively correlated with body weight, which was found to be decreased in the group following the low-fat vegan diet. The phylum Prevotella and Bacteroidetes also increased. The relative abundance of *Bacteroides fragilis* decreased in both groups, but less in the vegan group [99]. It has been shown that the phylum Bacteroidetes is three times less abundant in people with obesity than in normal-weight subjects. In addition, the abundance of Firmicutes has been described as increased in people with obesity [100]. The explanation could be provided by the finding that a 20% increase in Firmicutes and a corresponding decrease in the abundance of Bacteroidetes are associated with a 150 kcal/day increase in energy harvest, resulting in weight gain over time [101]. Therefore, an increase in the Bacteroidetes/Firmicutes ratio, as observed in a high-fiber, plant-based diet, can result in weight loss by reducing the number of calories extracted from the diet [22]. In the present study, however, despite an increase in Bacteroidetes after 16 weeks of a low-fat vegan diet, the ratio of Bacteroidetes to Firmicutes did not change in a statistically significant way. Therefore, a low-fat vegan diet induced significant changes in the gut microbiota, which were correlated with changes in body weight, suggesting potential use in clinical practice [99]. Certainly, more studies in patients with obesity are needed to further investigate the differences between the vegan and vegetarian dietary patterns on gut microbiota and weight loss potential (Table 6).

### 6.4. The Very Low-Calorie Ketogenic Diet

The Consensus Statement of the Italian Society of Endocrinology (SIE), published in 2019, states that VLCKD is a useful nutritional therapy to significantly changes the composition of the gut microbiota [38]. Despite this important role in modulating the gut microbiota, clinical studies on this effect are still scarce. In a recent study by Gutierrez-Repiso et al., the effect of a VLCKD on the gut microbiota of 33 subjects with obesity, with or without symbiotic supplementation, was evaluated. The protocol was composed of two phases: the first one consisted of 2 months of VLCKD with 600–800 kcal/day and a daily protein intake between 0.8–1.5 g/kg ideal body weight, and the second one consisted of two months of LCD (low-calorie diet) with 800–1500 kcal/day. Participants were randomized into three groups: (1) symbiotic supplementation in both the first and second phases of VLCKD; (2) placebo administration in the first phase and symbiotic supplementation in the second phase of VLCKD; (3) placebo supplementation in both the first and second phases of VLCKD. They observed that VLCKD without symbiotic supplementation resulted in a change in microbiota composition through the reduction of Proteobacteria and the increase in Firmicutes. As for families, Enterobacteriaceae, Sinobacteriaceae, and Comamonadaceae decreased, while the abundance of Ruminococcaceae and Morigibacteriaceae increased. Decreased Proteobacteria abundance has been seen to be positively correlated with reduced body weight and BMI [102]. An interesting pilot study conducted by Basciani et al. compared a whey protein-based VLCKD (*n* = 16, whey protein group WPG), a plant-based protein diet involving protein intake derived from soy, green peas, or cereals and a serving of low-glycemic index vegetables at lunch and dinner (*n* = 16, VPG vegetable protein group) and an animal protein diet involving protein intake derived from meat, fish, and eggs (*n* = 16, APG animal protein group), to investigate the effects on the gut microbiota in patients with obesity. The VLCKD was composed of 780 kcal/day, 26 g/day of carbohydrates (13.5% of total daily energy), 40.4% of total daily energy from fats (20 g of olive oil plus lipids from other sources), and about 90 g/day of proteins, about 1.2–1.4 g/kg (46.1% of total daily energy). After 45 days, in the VLCKD group, the relative abundance of Firmicutes and Actinobacteria significantly decreased, while that of Bacteroidetes and Proteobacteria significantly increased. At baseline, the abundance of Firmicutes and Bacteroidetes was almost overlapping in the three groups of patients. Over the weeks, regardless of diet type, the relative abundance of Bacteroidetes increased and that of Firmicutes decreased. The only exception was observed in the VPG, in which the increase in the abundance of Bacteroidetes did not reach statistical significance. The results showed that whey proteins and plant proteins were more potent in reducing Firmicutes abundance than VLCKD animal proteins. Among the three groups, WPG showed the greatest ability to increase Bacteroidetes. To summarize, the strongest effect of VLCKDs in reducing Firmicutes and increasing Bacteroidetes is that exerted by whey protein-containing VLCKDs compared with plant and animal protein-containing VLCKDs, but this result needs to be further studied in the long run [103] (Table 7).

## 7. Conclusions

**Figure 1:**
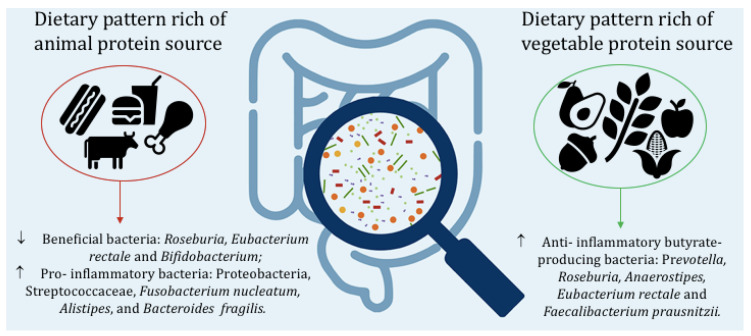
Effects of animal and vegetable protein on gut microbiota.

In recent years, the prevalence of obesity globally has grown rapidly. This has prompted researchers and health professionals to approach different dietary methods to achieve greater and healthier weight loss. Many dietary approaches have been considered. Increasing dietary protein content (compared with the recommended daily intake of 0.9 g/kg suggested by LARN guidelines [104]) has been one of the most intensively evaluated changes in macronutrient composition to manage body weight. However, it is also crucial to evaluate the quality of the suggested proteins. Indeed, the intake of animal protein sources such as beef, pork, fish, and eggs increase the intake of choline and carnitine, which are converted by the intestinal microbiota to trimethylamine and then oxidized in the liver and released into the circulation in the form of TMAO, which is strongly linked to cardiovascular risk [105]. Several studies have shown how an increase in grams of animal protein, double the recommended dietary allowance (RDA), can lead to a decrease in beneficial bacteria such as *Roseburia*, *Eubacterium rectale*, and *Bifidobacterium* [52,76,81,85,86] and an increase in *Bilophila*, *Alistipes*, *Bacteroides fragilis*, and *Bacteroides vulgates* [67,76,81]. In addition, the association of a high intake of animal protein with a higher intake of saturated fat, simple sugars, and fiber, typical of the Western diet, results in an increased inflammatory bacterial profile such as increased Firmicutes/Bacteroidetes, Proteobacteria, Streptococcaceae, *Fusobacterium nucleatum*, *Alistipes*, and *Bacteroides fragilis* ratios [69,72,81,91]. An important result, consisting of increased weight loss and stronger modulation of the relative abundance of Firmicutes and Bacteroidetes, was provided by a VLCKD with whey protein, underscoring the potential role of this animal protein source over others [103]. On the other hand, unsaturated fats, resistant starch, and diets rich in plant proteins, such as Mediterranean, vegetarian or vegan diets, lead to an important increase in butyrate-producing bacteria such as *Prevotella*, *Roseburia*, *Anaerostipes*, *Eubacterium rectale*, and *Faecalibacterium prausnitzii* [60,72,75,94,95,97,98,99]. The commensal genus Prevotella belongs to the phylum Bacteroidetes, which is hypothesized to be related to higher consumption of carbohydrates, fiber, and plant proteins: food components characteristic of the Mediterranean diet [97]. Subjects with greater adherence to a Mediterranean diet showed greater bacterial diversity and gene richness, a relative abundance of beneficial and anti-inflammatory bacteria [98,99], and a reduction in the endotoxigenic *Bacteroides fragilis* [99] suggested to be associated with acute and persistent diarrheal disease, IBD, and colorectal cancer [106]. In addition, the relative abundance of *Faecalibacterium prausnitzii* is directly correlated with reduced body weight [107]. Therefore, a dietary pattern that promotes the growth of this beneficial commensal is necessary. Therefore, healthy diets rich in fiber and plant protein and with adequate amounts of fat, carbohydrate, and animal protein can help beneficially modulate host–microbe interactions and identify effective pathways involved in weight loss and disease prevention (Figure 1). Studies in the literature often do not distinguish between animal and plant protein intake but consider the total percentage of daily caloric intake derived from protein. Therefore, it is not possible to analyze well and in depth the substantial difference between the two protein sources and their different effects. Further studies are needed to compare the different effects of a plant or animal protein diet on the human gut microbiota in overweight or with obesity subjects.

## Figures and Tables

**Table 1:** Effect of carbohydrates on gut microbiota.

Study	Population	Dietary Effects	Results
Costabile et al., 2008 [55]	32 healthy volunteers with BMI between 20 and 30 kg/m^2^	Consumption of either 48 g of whole grain (WG) or wheat bran (WB) for two 3-week periods, separated by a 2-week washout period	WG: ⇑ Bifidobacteria and lactobacilli No differences in SCFAs between the two groups
Cotillard et al., 2013 [52]	38 with obesity and 11 overweight subjects	12 weeks of energy-restricted high protein diet with 35% protein, 25% lipids, and 44% carbohydrates	Dietary intervention: ⇑ gene richness ⇓ Bifidobacterium and *Eubacterium rectale*.
Birkeland et al., 2020 [58]	25 subjects with BMI < 40 kg/m^2^ affected by type II diabetes	16 g of inulin-type fructans (a mixture of oligofructose and inulin) and 16 g placebo (maltodextrin) for 6 weeks in randomized order	The inuline-type fructans: ⇑ *SCFAs* ⇑ *Bifidobacterium adolescentis*
Sheflin et al., 2017 [59]	29 overweight and with obesity volunteers	Consumption of a snack with fiber-rich, stabilized rice bran (30 g/day), or cooked navy bean powder (35 g/day) for 28 days	Supplementation with rice bran: ⇓ the Firmicutes to Bacteroidetes ratio ⇑ SCFAs
Walker et al., 2011 [60]	14 overweight men	Volunteers were provided successively with a control diet, diets high in resistant starch (RS) (26 g/day of resistant starch from type III resistant starch), or non-starch polysaccharides (NSPs) (42 g/day of total non-starch polysaccharides from wheat bran) and a reduced carbohydrate weight loss (WL) diet over 10 weeks	RS diet: ⇑ *Ruminococcus bromii* (17%) of total bacteria compared to the 3.8% of the NSP diet ⇑ *Eubacterium rectale* (10.1%) RS and WL diets: ⇑ *Oscillibacter* WL: ⇓ *Eubacterium* and *Collinsella aerofaciens*
Abell et al., 2008 [63]	46 overweight volunteers	One group received 4 weeks of a RS2—rich diet (22 g/day of RS) and the other one received 4 weeks of a RS2—low diet (1 g/day of RS).	The RS2-rich diet group showed ⇑ *Ruminococcus bromii*, *Faecalibacterium prausnitzii* and *Eubacterium rectale*

Legend: ⇑ increase; ⇓ decrease.

**Table 2:** Effect of fats on gut microbiota.

Study	Population	Dietary Effects	Results
Wan et al., 2019 [69]	217 overweight subjects	6 months of either a high-fat (40%) or a low-fat (20%) diet	The high-fat diet: ⇑ *Alistipes*, *Bacteroides* and the LPS ⇓ *Faecalibacterium* and total SCFAs
Fava et al., 2013 [66]	88 overweight subjects	Low-fat (28% fat) diet and a high saturated fat (38% fat of which 18% was from saturated fats) diet for 4 weeks	Low-fat diet: ⇑ fecal abundance of *Bifidobacterium* High saturated fat diet: ⇑ *Faecalibacterium prausnitzii*
Rajkumar et al., 2014 [70]	60 overweight adults	1 capsule daily of omega-3 polyunsaturated (PUFA) supplementation (containing 180 mg of EPA and 120 mg of DHA per capsule) for 6 weeks	Did not lead to a significative change on gut microbiota composition
Balfegò et al., 2016 [71]	35 overweight and with obesity patients	One group followed a standard diet and the other one a diet enriched with 100 g of sardines for 5 days/week for 6 months	The group that followed the enriched diet, compared to the baseline showed: ⇓ in the Firmicutes to Bacteroidetes ratio ⇑ in the Bacteroides to Prevotella ratio
Jian et al., 2021 [72]	38 overweight and with obesity subjects	Excess of 1000 kcal/day in a diet rich in either saturated (86% of total daily energy from fat, of which 76% were saturated) or unsaturated fats (91% of total daily energy from fat, of which 79% were unsaturated)	Overfeeding of saturated fats: ⇑ Proteobacteria Overfeeding of unsaturated fats: ⇑ butyrate producers bacteria
Vijay et al., 2021 [73]	69 subjects who had a BMI comprised between 20 and 39.9 kg/m^2^	6-week dietary intervention with a supplementation of either 500 mg of omega-3 or 20 g of inulin	The omega-3 supplementation resulted in: ⇑ *Coprococcus* spp. and Bacteroides spp. ⇓ *Collinsella* spp (fatty-liver associated)
Bratlie et al., 2021 [74]	76 adults with overweight/obesity	Intake of salmon, cod (both in the quantity of 750 g/week) or the total exclusion of fish (control group) for 8 weeks	Salmon and Cod groups determined: ⇓ abundance of Bacteroidetes, Clostridiales; ⇑ abundance of the Selenomonadales.
Telle-Hansen et al., 2022 [75]	17 humans with a BMI between 18.5 and 27 kg/m^2^	Consumption of products with saturated fatty acids (SFAs) (29.9 g of SFAs/day) or polyunsaturated fatty acids (PUFAs) (26.4 g of PUFAs/day) for 3 days	The intervention with PUFAs: ⇑ Lachnospiraceae and *Bifidobacterium* spp.

Legend: ⇑ increase; ⇓ decrease.

**Table 3:** Effect of proteins on gut microbiota.

Study	Population	Dietary Intervention	Results
Beaumont et al., 2017 [80]	38 overweight subjects	3 weeks of 15% of total daily energy supplementation with soy protein, casein, or maltodextrins as control	No significant differences in bacterial composition between the intervention groups but supplementation (of soy or casein) can increase the luminal concentrations of both beneficial and deleterious compounds
Hentges et al., 1977 [81]	10 volunteers	4 months of high beef diet with 176 g/day of proteins (double content of proteins compared to the meatless diet) vs. meatless diet with 90 g/day of protein	The high beef diet: ⇓ *Bifidobacterium adolescentis* ⇑ *Bacteroides fragilis* and *vulgatus*
Romond et al., 1998 [82]	20 volunteers	Consumption of twice daily for 7 days 15 mL of concentrated whey from milk fermented with *Bifidobacterium breve*	⇓ *Bacteroides fragilis* and *Clostridium perfringens*
Sun et al., 2022 [83]	60 overweight or with obesity older women	Supplementation for 8 weeks of 15.2 g/day of whey protein or 16.8 g/day of whey protein hydrolysate to the basal quantity of 50 g/day of proteins	No significant changes in gut microbiota composition
Reimer et al., 2017 [84]	125 adults with overweight/obesity	Consumption of 2 isocaloric snack bar/day with 5 g of whey protein for 12 weeks	No variation of gut microbiota
Mitchell et al., 2019 [85]	31 healthy older men with BMI between 20 and 35 kg/m^2^	Consumption of the recommended dietary allowance of protein (RDA: 0.8 g protein/kg bodyweight/day) or twice the RDA (2RDA) as part of a supplied diet for 10 weeks	No significant differences in proteolytic microbiota or metabolites of protein fermentation
Russell et al., 2011 [86]	17 men with obesity	High protein (137 g/daily)/low carbohydrate (22 g/daily) diet for 4 weeks	⇓ *Roseburia*, *Eubacterium rectale* and butyrate
Clarke et al., 2014 [77]	40 overweight professional rugby athletes and 46 controls	The athletes usually consumed higher quantities of protein (22% of daily energy)	⇑ gut microbiota diversity and *Akkermansia* compared to controls

Legend: ⇑ increase; ⇓ decrease.

**Table 4:** Effect of Western diet on gut microbiota.

Study	Population	Dietary Intervention	Results
O’keefe et al., 2015 [91]	20 subjects African Americans and 20 rural Africans with a BMI range between 18–35 kg/m^2^	African Americans were fed a high-fiber (55 g/day), low-fat (16% energy), and 14% energy from protein African-style diet, and rural Africans a high-fat (52% energy) low-fiber (12 g/day), and 27% energy from protein Western-style diet for 2 weeks	The switch to the high-fat/low-fiber diet: ⇑ *Fusobacterium nucleatum* The switch to the low-fat/high fiber diet: ⇓ *Bilophila wadsworthia*
Cotillard et al., 2013 [52]	38 with obesity and 11 overweight subjects	12 weeks of energy-restricted high protein diet with 35% protein, 25% lipids, and 44% carbohydrates	Dietary intervention: ⇑ gene richness ⇓ Bifidobacterium and *Eubacterium rectale*.
Reddy et al., 1975 [67]	8 healthy adults	Consuming 4 weeks of high meat mixed Western diet, consisting in a consumption of 454 g/day of beef, pork, lamb, and/or chicken (23% protein, 45%fat, and 32% carbohydrate) and then switching to a nonmeat diet (20% protein, 30% fat, and 50% carbohydrate).	During the period of consumption of a high meat mixed Western diet compared to the nonmeat diet: ⇑ Bacteroides, Bifidobacterium, Peptococcus, and anaerobic Lactobacillus
David et al., 2014 [76]	10 subjects with BMI range between 19 and 32 kg/m^2^	5 days of an animal-based diet composed of meat, eggs and cheeses (69.5% fat, 30.1% protein, and fiber intake nearly zero) compared to a plant-based diet composed of grains, legumes, fruits, and vegetables (22% fat and 10% protein and 25.6 g fiber/1000 kcal)	The animal-based diet: ⇑ the abundance of bile-tolerant microorganisms (*Alistipes*, *Bilophila*, and Bacteroides) ⇓ the levels of Firmicutes that metabolize dietary plant polysaccharides (*Roseburia*, *Eubacterium rectale*, and *Ruminococcus bromii*)

Legend: ⇑ increase; ⇓ decrease.

**Table 5:** Effect of Mediterranean diet on gut microbiota.

Study	Population	Dietary Intervention	Results
Rinott et al., 2022 [94]	294 participants with visceral obesity	Three intervention groups: (1) healthy dietary guidelines (standard science-based nutritional counseling), (2) MED (3) Green-MED. Both isocaloric MED and Green-MED groups were rich in vegetables, with poultry and fish replacing beef and lamb intake, with a restriction of processed and red meats, a consumption of 28 g of walnuts, and with a 40% of total daily energy from mainly unsaturated fat, a daily protein intake of 123 g on average and a daily carbohydrates intake of 80 g. The Green-MED group was further provided with daily polyphenol-rich green tea and Mankai aquatic plant.	Both MED and Green-MED induced to modification of but microbiota. The Green-MED led to ⇑ *Prevotella* and enzymatic functions involved in branched-chain amino acid degradation, ⇓ *Bifidobacterium* and enzymatic functions responsible for branched-chain amino acid biosynthesis
Pagliai et al., 2020 [95]	23 overweight/with obesity omnivores	Randomly assigned to follow for 3 months either a Vegetarian diet (VD) or a Mediterranean diet (MD) with 50–55% of total daily energy from carbohydrate, 25–30% from fat and 15–20% from proteins each	No significantly differences between the two diets at ranks such as phyla and families; VD significantly resulted in ⇑ *Anaerostipes* and *Streptococcus* ⇓ *Clostridium*, *Odoribacter* and Propionate production MD significantly resulted in ⇑ *Lachnoclostridium* and *Enterohabdus* ⇓ *Parabacteroides*
Gutierrez-Diaz et al., 2016 [97]	31 overweight adults	The group with high adherence to Mediterranean diet (≥4) was characterized by 48 g/day of proteins (of which 32 g/day are from animal source and 15 g/day are from vegetable one), 103 g/day of carbohydrates, and 40 g/day of fats, compared to a 51 g/day of proteins (of which 37 g/day are from animal source and 13 g/day from vegetable one), 96 g/day of carbohydrates, and 43 g/day of fats of the lower adherence group	MDS ≥ 4 was associated with ⇑ Bacteroidetes, Prevotellacea and Prevotella, fecal propionate and butyrate. ⇓ Firmicutes and Lachnospiraceae
Meslier et al., 2020 [98]	82 overweight and with obesity subjects	43 subjects followed an isocaloric Mediterranean diet (MD) for 8 weeks with a total amount of about 63 g/day of proteins (of which 37 g/day are from vegetable source and 26 g/day from animal source) and 39 followed their habitual diet with low intake of vegetable and fruits (control group) with a total amount of about 68 g/day of proteins (of which 20 g/day are from vegetable source and 48 g/day are from animal one)	The MD intervention leads to: ⇑ levels of the fibre-degrading *Faecalibacterium prausnitzii* and of genes for microbial carbohydrate degradation linked to butyrate metabolism.

Legend: ⇑ increase; ⇓ decrease.

**Table 6:** Effect of vegetable protein rich diets on gut microbiota.

Study	Population	Dietary Intervention	Results
Pagliai et al., 2020 [95]	23 overweight/with obesity omnivores	Randomly assigned to follow for 3 months either a Vegetarian diet (VD) or a Mediterranean diet (MD), with 50–55% of total daily energy from carbohydrate, 25–30% from fat, and 15–20% from proteins each	No significantly differences between the two diets at ranks such as phyla and families; VD significantly resulted in: ⇑ *Anaerostipes* and *Streptococcus* ⇓ *Clostridium*, *Odoribacter* and propionate production MD significantly resulted in: ⇑ *Lachnoclostridium* and *Enterohabdus* ⇓ *Parabacteroides*
Kahleova et al., 2020 [99]	168 overweight participants	84 subjects followed for 16 weeks a low-fat vegan diet (with 43 g/day of proteins, of which 1 g/day come from animal source and 42 g/day from vegetable one and 24 g/day of fats) and 84 subjects followed a control diet (with 69 g/day of proteins, of which 39 g/day come from animal source and 29 g/day from vegetable one and 72 g/day of fats)	After 16 weeks of low-fat vegan diet: ⇑ Increased Bacteroidetes, *Faecalibacterium prausnitzii* and Prevotella ⇓ *Bacteroides fragilis*

Legend: ⇑ increase; ⇓ decrease.

**Table 7:** Effect of VLCKD on gut microbiota.

Study	Population	Dietary Intervention	Results
Gutierrez-Repiso et al., 2019 [102]	33 patients with obesity	2 months of VLCKD (daily protein intake between 0.8–1.5 g/kg ideal body weight) and then 2 months of a LCD. Subjects were randomly allocated to three groups: (1) supplemented with symbiotics, (2) supplemented with a placebo during the VLCKD and symbiotics during the LCD phase, and (3) placebo for the control group	VLCKD without the supplementation of symbiotics resulted in: ⇓ Proteobacteria and Enterobacteriaceae, Sinobacteraceae and Comamonadacea ⇑ Firmicutes, Ruminococcaceae and Morigibacteriaceae.
Basciani et al., 2020 [103]	48 patients with obesity randomized in 3 groups of 16 subjects each	45 days one of these 3 different VLCKD (all composed of 26 g/day of carbohydrates, 40.4% of total daily energy from fats and about 90 g/day of proteins, about 1.2–1.4 g/kg): (1) Whey protein VLCKD (WPG) (2) Vegetable protein VLCKD (VPG) (3) Animal protein VLCKD (APG)	Independently from the VLCKD type: ⇑ Bacteroidetes; ⇓ Firmicutes WPG determined the strongest effect in: ⇓ *Firmicutes* ⇑ *Bacteroidetes* compared to VPG and APG.

Legend: ⇑ increase; ⇓ decrease.

## Data Availability

Not applicable.
